# Total knee arthroplasty coronal alignment and tibial base stress—a new numerical evaluation

**DOI:** 10.1097/j.pbj.0000000000000208

**Published:** 2023-04-10

**Authors:** João Vale, Luisa V. Pinto, Bianca Barros, Sara Diniz, Filipe Rodrigues, Marco Marques, Jorge Belinha, Adélio Vilaça

**Affiliations:** a Department of Orthopaedics, Centro Hospitalar do Universitário do Porto, Porto, Portugal; b Department of Physical and Medicine Rehabilitation, Centro Hospitalar de Entre o Douro e Vouga, Santa Maria da Feira, Portugal; c Institute of Science and Innovation in Mechanical and Industrial Engineering, INEGI, Porto, Portugal; d Department of Mechanical Engineering, School of Engineering, Polytechnic of Porto, ISEP, Porto, Portugal

**Keywords:** total knee arthroplasty, stress shielding, bone remodeling, finite element method, meshless methods, total knee arthroplasty coronal alignment

## Abstract

**Background:**

Total knee arthroplasty (TKA) is one of the most frequently performed orthopedic procedures. The correct positioning and alignment of the components significantly affects prosthesis survival. Considering the current controversy regarding the target of postoperative alignment of TKA, this study evaluated the tension at tibial component interface using two numerical methods.

**Methods:**

The stress of the prosthesis/bone interface of the proximal tibial component was evaluated using two numerical methods: the finite element method (FEM) and the new meshless method: natural neighbor radial point interpolation method (NNRPIM). The construction of the model was based on Zimmers NexGen LPS-Flex Mobile® prosthesis and simulated the forces by using a free-body diagram.

**Results:**

Tibiofemoral mechanical axis (TFMA) for which a higher number of nodes are under optimal mechanical tension is between 1° valgus 2° varus. For values outside the interval, there are regions under the tibial plate at risk of bone absorption. At the extremities of the tibial plate of the prosthesis, both medial and lateral, independent of the alignment, are under a low stress. In all nodes evaluated for all TFMA, the values of the effective stresses were higher in the NNRPIM when compared with the FEM.

**Conclusion:**

Through this study, we can corroborate that the optimal postoperative alignment is within the values that are currently considered of 0 ± 3° varus. It was verified that the meshless methods obtain smoother and more conservative results, which may make them safer when transposed to the clinical practice.

## Introduction

Total knee arthroplasty (TKA) is one of the most frequently performed orthopedic procedures, with an average of 118 primary procedures per 100,000 habitants, according to the Organization for Economic Cooperation and Development.^1^ These numbers have been increasing, and it is estimated that the need for TKAs performed annually will increase 673% by 2030 in the United States.^2^ These values are due to an increase in the number of people suffering from osteoarthrosis, justified by both the aging population and the prevalence of obesity, which is the main risk factor after age and sex.^3^

TKA has a significant impact in quality of life, especially regarding pain relief and function improvement. However, some fail during the expected survival, requiring surgical revision.^4^ With the increase in TKA, it is estimated that the number of revisions also increases.^5^ It is important to improve the survival of the prosthesis because the revisions are associated with less satisfactory results and an increased risk of complications.^4^

The variation of prosthetic survival is associated with differences in implant design, fixation type, indication for TKA, surgical technique, and patient-related factors.^6^ Regarding the surgical technique, it is widely accepted that the correct positioning and alignment of the components is one of the factors that significantly affects prosthesis survival and patient satisfaction.^7^ In fact, it is reported as the first cause of revision in 2.9%–7% of total revisions of TKA,^8,9^ having a role in early reviews (up to 5 years) and with an even more significant impact together with instability, accounting for 27% of revisions.^9^ By itself, the appropriate alignment of TKA is related to greater stability, lower release rate, and higher clinical scores.^10^ Thus, the restoration of a suitable mechanical axis is one of the goals of the TKA, and it is considered the most important factor managed by the surgeon.^11^ Despite its importance, the current literature does not have values of reference for alignment in the coronal plane.^10^

Alignment can be described in two ways: the tibiofemoral anatomical angle and the tibiofemoral mechanical angle (TFMA). Traditionally, the safety zone values of 0 ± 3° varus of the TFMA have been used to define correctly aligned versus malaligned knees.^12^ Most studies have shown an increase in revision rates with poor alignment in the coronal plane.^10,11,13-15^ However, recent studies show a small correlation between these.^16-18^ Recently, Bellemans et al found that 32% of men and 17% of women had varus knee, introducing the concept of constitutional varus and questioning the benefits of a neutral alignment in these patients.^19^

Considering the current controversy regarding the target postoperative alignment of TKA, this study will use two numerical methods: the traditional and widely used Finite Element Method (FEM) with a new meshless method: Natural Neighbor Radial Point Interpolation Method (NNRPIM), comparing the results regarding the stimulation of bone adaptation at the prosthesis/knee interface.

Meshless methods are seen as the next generation of computational techniques, receiving increased attention from researchers, due to the limitation of conventional methods based on fixed meshes, such as the FEM.^20^ NNRPIM is an organic method and does not require a structured mesh. Thus, NNRPIM is capable to perform the computational analysis directly using the voxels spatial information from a computed tomography (CT) or a magnetic resonance imaging. Comparing with other numerical approaches, NNRPIM allows producing smoother and more accurate variable fields, such as displacement and strain/stress.^21^

With the use of these two numerical methods, the TKA/bone interface tension will be evaluated, trying to find an optimal range of TFMA, to select the best clinical solution for the patient.

## Methods

### Material

In this study, the stress of the prosthesis/bone interface of the proximal tibial component was evaluated. To simplify, it was defined that the tibial component of the total knee prosthesis was aligned with the mechanical axis of the tibia. Thus, only the variation of the placement of the femoral component would affect the stress to which the proximal tibial bone would be subjected. The construction of the model was based on Zimmer®'s NexGen LPS-Flex Mobile prosthesis.

### Model construction

For the construction of the model, the three components involved in the tibial component of the knee prosthesis were considered: proximal tibial bone, tibial part of the prosthesis (CoCrMo metal component and mobile component of UHMWPE), and bone cement. Initially, the 3D model of the bony component of the proximal tibia was constructed using the Mimics® commercial program, based on a CT. The commercial 3-matic® software was subsequently used to make adjustments to the resulting model, both of Materialise® software.

For the construction of the tibial portion of the prosthesis, the size of the prosthesis was initially defined. It was used as a measurement, the calibration of the medial-lateral size of the tibial plate by CT scan (79.6 mm), which corresponded to a prosthesis size number 8. After size selection, the metal component of CoCrMo and UHMWPE was constructed through commercial software Solidworks®, based on Zimmer® molds.

At last, the metal component of the prosthesis was placed in the 3D model of the proximal tibial bone. For its placement, a cut was made perpendicular to the mechanical axis of the tibia, 9 mm below the articular interline, mimicking the surgical technique.

A layer of thickness of 3 mm^22^ was designed and placed on the tibial plate, to simulate the layer of cement introduced during the surgical procedure.

**Figure 1.**
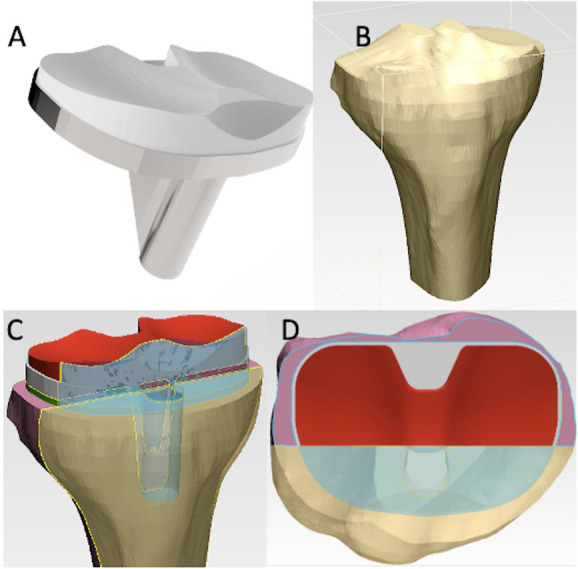
(A) Prosthesis 3D model; (B) proximal tibia 3D model; (C) 3D model construct; and (D) coronal plane level.

To obtain the 2D geometric model, intended to be used in the numerical study, a coronal cut was performed on the 3D model constructed. The selected cut plane took into account the mean anterior-posterior plane, and slightly anterior positioned one to the latter was selected, aiming to incorporate the stem into the 2D model. After obtaining the 2D model to be tested (Fig. 1), the problem (generating a mesh of nodes and finite elements) was smoothened using FEMAP® software (student version).

### Material constants definition

For the definition of the material constants, we used the highest reference value for the elasticity module, and the lower value for the Poisson's coefficient found in the literature, for all the constituents of the model (see Appendix A, Supplemental Digital Content 1, http://links.lww.com/PBJ/A29).

### Application of forces

**Figure 2.**
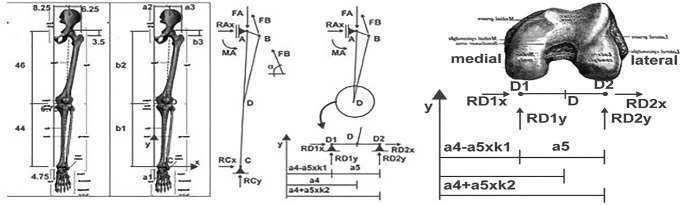
Free body used to calculate the force at which the proximal tibia would be subjected to different knee alignments.

To calculate the force at which the proximal tibia would be subjected to different knee alignments, we used the static fundamental equations, assuming the free-body diagrams presented and based on Fig. 2. It is possible to find in the literature some related works.^23-25^ The geometric parameters shown in Fig. 2 are presented in Supplemental Digital Content 2 (Appendix B, http://links.lww.com/PBJ/A29).

In the calculation, two variables were considered: k1 and k2, where k1 + k2 = 1, in which k1 corresponds to the deviation percentage from the midtibiofemoral to medial contact point. It was considered that the value of k1 varies only between 0 and 1 because outside these limits would have to take into account the role of collateral ligaments, a role that was not considered in this study. The model was simplified considering that for a tibiofemoral mechanical angle (TFMA) = 0° in varus, the knee is normoaligned and, thus, with the value of k1 = k2. At a value of k1 corresponds a value of the TFMA (Table 1). Like k1, TFMA also has limits, being they ±6.3° in varo. Thus, for values of k1 = k2, k1> k2, and k1 <k2 corresponds, respectively, a normoaligned knee, valgus, and varus.

**Table 1:** Application of forces.

		K1=0, TFMA= 6.3°	K1=0.14, TFMA=4.5°	K1=0.26, TFMA=3°	K1=0.5, TFMA=0	K1=0.74, TFMA=-3°	K1=0.86, TFMA=-4.5°	K1=0,93, TFMA=-6.4°
Medial force (N)	x	72,5	61.7	52.4	33.9	15.4	6.2	-4.6
	y	1594,5	1357	1153.5	746.4	339.3	135.7	-101.8
Lateral force (N)	x	4.6	15.4	24.7	43.2	61.7	70.9	81.7
	Y	101.8	339.3	542.8	949.9	1357	1560.6	1798.1

### Results

To perform both FEM and NNRPIM analyses, FEMAS® academic software (cmech.webs.com) was used. The constructed prosthesis/bone interface 3D model was evaluated, comparing the FEM and NNRPIM results.

### Stress calculations

The stress field of the 2D model was obtained with FEMAS. Several stress fields were obtained for the different values of k1, which correspond to different TFMA values (Table 1).

It was considered a joint contact area of 150 mm^2^ (10 x 15) in each condyle,^26^ considering the simplified two-dimensional analysis, taking into account a flat state of deformation (granting a slice of material of only 1 mm). The load applied in Table 1 should be applied considering a ratio of 1/15.

### Statistical analysis

**Figure 3.**
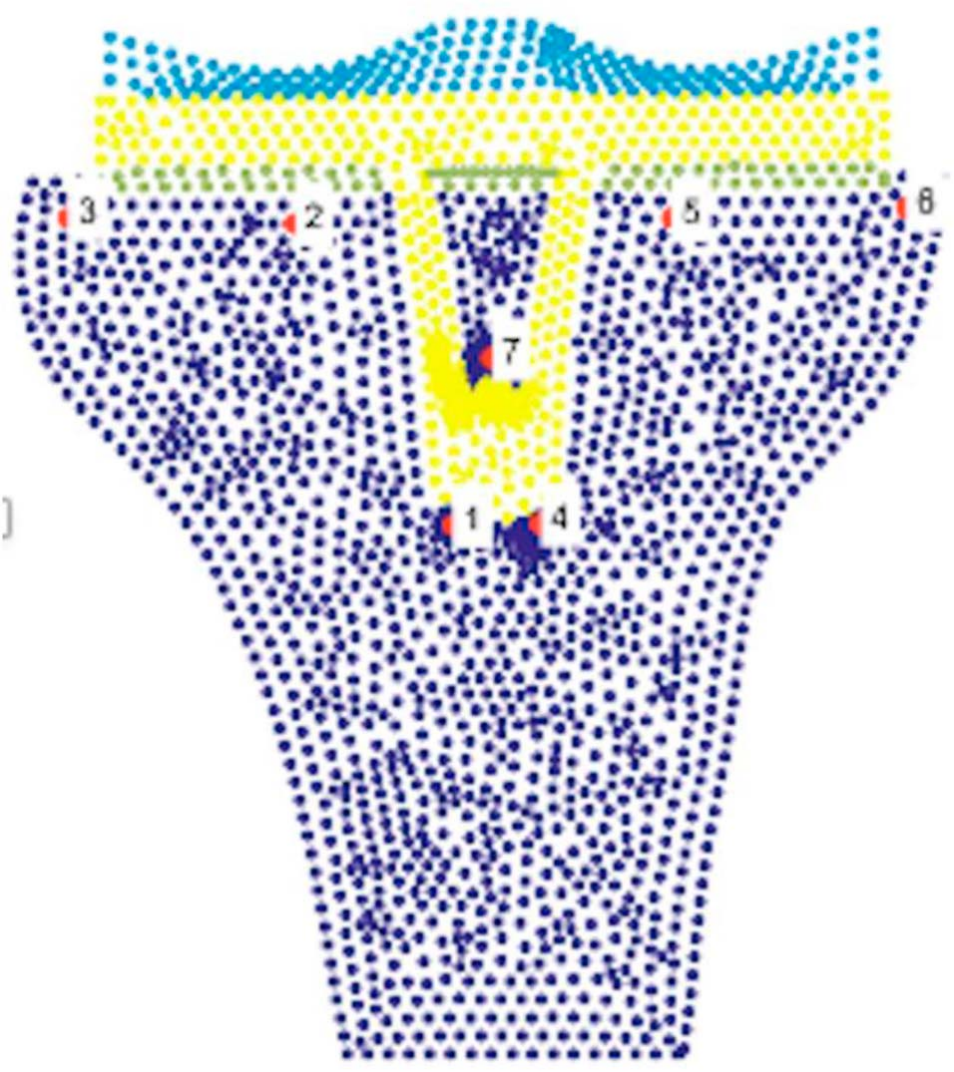
Legend and location of selected knots under study

Initially, we used a qualitative evaluation of the stresses to which the model would be a subject to different angles. This analysis allows the selection of the node that would be subject to more or less stress, according to the risk of formation and bone resorption, respectively. (Fig. 3).

A quantitative evaluation of the 7 selected nodes (2 in the medial plate, 2 in the lateral plate, and 3 perirod) was followed. For this quantitative evaluation, the effective stress at which each node was subjected was evaluated, varying the TFMA. This variation was evaluated with two numerical methods, FEM and NNRPIM, and represented in graphs, one for each method.

To evaluate the differences between the numerical methods, a qualitative evaluation was carried initially by comparing the images obtained after simulation, for the same value of TFMA. Then, the percentage of stress variation at a given point was evaluated quantitatively, taking into account their value, in the neutral alignment (TFMA = 0°). After obtaining this value, the variation of the 7 nodes under study at each angle was averaged, and its variation by AMTF in each method was plotted.

## Results

### Qualitative analysis

**Figure 4.**
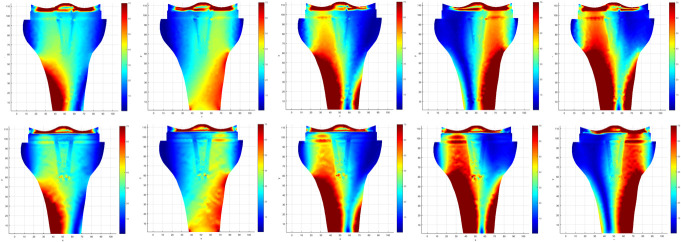
Stress maps in different alignments (-6,3; 3; 0; 3; 6,3° in varus from left to the right, respectively), using two numerical methods FEM and NNRPIM, in the upper and lower rows, respectively.

Stress maps were obtained with the two numerical methods used for the 7 TFMA values tested (Fig. 4). Through the qualitative analysis of the prosthesis/bone interface, it is observed that NNRPIM provides highly detailed stress distributions. Through the interpretation of the color scale, as a rule, the NNRPIM method presents higher stresses than the FEM.

The analysis of the stress map of the two methods, for different values of TFMA presented in Fig. 4, allowed to select the nodes presented in Fig. 3. These selected nodes were those that qualitatively were in zones of higher or lower stress and, therefore, under the risk of bone formation or resorption, respectively. An exception was where the nodes were at the interface with the prosthesis stem (1, 4, and 7), in which, although qualitatively they did not encounter high stresses, they were selected to represent the stress values to which this region is subject.

### Quantitative analysis

The literature^23^ indicates that for tension values lower than 2 MPa, the bone tissue initiates the absorption process, and for values above 2 MPa, bone remodeling occurs. Thus, the ideal TFMA will be the one where most of the points are close to this value and, as such, are neither at risk of resorption nor over training.

**Figure 5.**
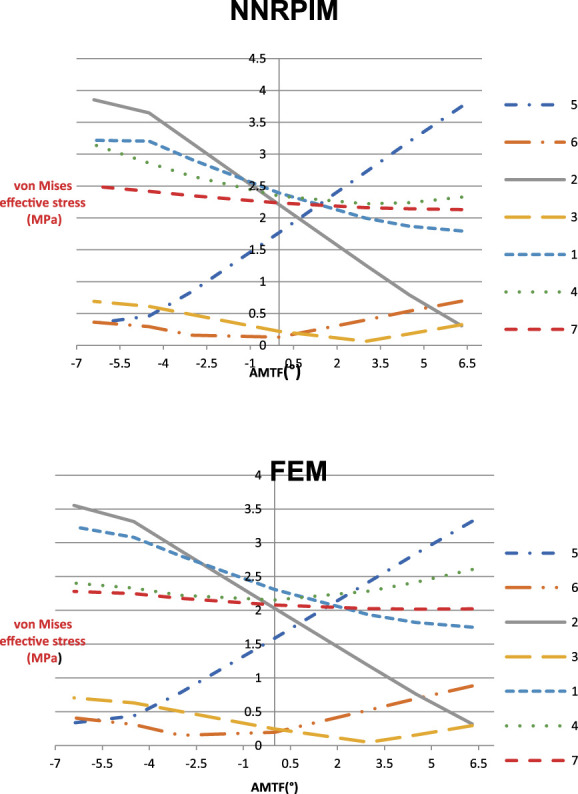
Variation of the effective tension of the selected nodes, varying the value of TFMA, in the NNRPIM and FEM.

The results obtained in FEM and NNRPIM were represented in graphs, in which the von Mises effective stress of the 7 nodes selected varies with the TFMA values (Fig. 5).

Considering the premise that values that are far apart from 2 MPa are subject to absorption (lower values) or bone formation (higher values), it is observed that the TFMA for which a higher number of nodes are close to 2 MPa corresponds to the interval between 1st valgus and 2nd varus. The TFMA value in which most of the population is closer to 2 MPa is the 2nd in varus.

When performing the individual evaluation of the nodes, it can be observed that there are two nodes (6 and 3) that, regardless of the value of the TFMA, are always at risk of bone absorption because they present values of von Mises effective stress lower than 1 MPa.

In the analysis of the peristem nodes (1, 4, and 7), it is observed that these do not suffer significant stress variation with the TFMA variation, always remaining close to the value of 2 MPa. However, node 1, which is located at the bone interface, in contact with the lower end of the prosthesis shaft, is subject to higher stresses as the TFMA value approaches a valgus position.

On the contrary, points 2 and 5 are those that suffer the most significant change in the von Mises effective stress values. These points also have symmetrical behaviors because as one increases, the other decreases. For TFMA values higher than 2 are at risk of absorption (point 2) and formation (point 5) and, for values of TFMA lower than -1, at risk of absorption (point 5) and formation (point 2).

The graphs obtained show very similar values for the same points, regardless of the method used.

**Figure 6.**
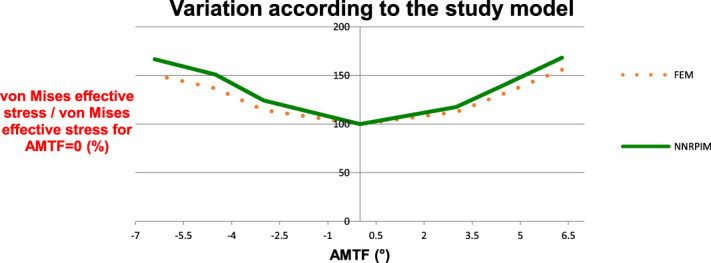
Evaluation of differences between numerical methods.

Among the methods, it is observed that there is only a variation in the stress magnitude. Figure 6 shows a similar behavior for all models. As variations of the TFMA values occur, it can be observed that, regardless of the value of TFMA, meshless method presents higher values than FEM.

The mean values of the ones presented in Fig. 6 were evaluated at each point between the meshless method and the FEM method. Among these, it was observed that the NNRPIM had 6.5% higher values on average.

## Discussion

Bone is a dynamic, metabolically active living tissue that is constantly growing, remodeling, and repairing itself throughout life to maintain stability and integrity.^27^ Remodeling requires cells that reabsorb the old bone, the osteoclasts, and the cells that form new bone and the osteoblasts. The ability of the bone to adapt to mechanical loads is also based on reabsorption and bone formation performed by bone cells. In the process of adaptation, usually referred as bone remodeling, reabsorption and bone formation occur at different sites, with a consequent alteration of the bone morphology. To adapt to mechanical conditions, bone remodeling also requires cells sensitivity to mechanical stimuli, the osteocytes.^23^

The correlation between loads and bone adaptation is described in Wolf law, which states that forces are felt by the bone and it adapts its structure and morphology according to the mechanical stimulus, acquiring a more resistant structure to that load, always trying to minimize its mass.^28,29^ Mechanical stimuli are a strong stimulus for the bone, leading to bone adaptation, allowing its tissue to adjust to the originated stresses.^30^

Considering the importance of the mechanical stimulus in the bone properties, namely structure and morphology, stress shielding effect must be taken into account when there is an implant placement. Stress shielding is the redistribution of stress in the bone at the time of implant placement and is one of the mechanical factors that may contribute to implant failure in the bone. It arises as a consequence of the reduction of the mechanical load on the bone by the presence of an adjacent implant, resulting in bone loss and atrophy (resorption). Bone resorption may lead to loss of mechanical support and/or bone stock, implant migration, and fracture of the prosthesis and/or bone.^23^

As expected, given the importance of mechanical effects on bone adaptation, the metaphysis of the tibial bone is able to adapt to mechanical changes, such as poor alignment caused by osteoarthrosis.^31^ After placement of the knee implant, the bone of the metaphysis also adapts to the changes of loads, after correcting the preoperative misalignment.^32^ Bone remodeling that occurs due to a change in load may cause the periprosthetic bone to undergo stress shielding because there is a decrease in local stress, as it is partially transferred through the cement and prosthesis.^33^ In addition, the bone density was shown to be closely correlated with the mechanical properties of the bone, suggesting that the loss of bone due to the tibial component of the prosthesis is a major concern to the success of the TKA, since it may be a threat to knee stability.^34^ Thus, the understanding of the bone adaptation process, taking into account the mechanical behavior, is an important issue, especially in the selection of the most suitable orthopedic implant.^35^

Considering the importance of the mechanical stimulus after implant placement in the bone adaptation, this study evaluated the variation of stress in the periprosthetic region, varying the alignment of the prosthesis. It was observed that the values of TFMA between 1° valgo and 2° in varo are those that present values closer to 2 MPa, in which a balance between bone formation/absorption occurs. This value is in agreement with what is currently being sought during the TKA, which is the imposition of the position of the normoaligment prosthesis (TFMA = 0 ± 3° varus).^12^ It is also in agreement with the results obtained in several studies, which show an increase in surgical revision rates for values outside this range.^10,11,13-15^ In this study, it is observed that for values outside of the interval between 1st and 2nd values, there are regions under the tibial plate, namely those represented by knots 2 (lateral region) and 5 (medial region), which are at risk of bone absorption, probably due to stressing effect, which risks the stability of the prosthesis and, consequently, increases the risk of failure and surgical revision.

It was also observed that independent of the alignment, the extremities of the tibial plate of the prosthesis, both medial (node 6) and lateral (node 3), are regions under a very low stress and consequently at risk of bone absorption. This effect is probably due to the process of stress shielding, in which the design of the prosthesis redistributes the stresses, sparing these regions, making them susceptible to bone absorption and jeopardizing prosthetic stability. In this way, the extremities should be considered as regions at risk of bone absorption, which should be taken in consideration when selecting the prosthesis.

As for the nodes located in the perirod region (1, 4, and 7), we can observe that they have relatively constant effective stress values, independently of the alignment. These data are in agreement with what is currently the standard surgically, which is the use of prosthesis with short rods. When compared with the larger stems, the latter are associated with higher values of periprosthetic bone resorption and higher stress concentrations at the end of the stem, with a consequent greater risk of failure.

To obtain the results, the meshless methods NNRPIM and FEM were used.^36^ In all nodes evaluated for all TFMA values, it was found that the values of the effective stresses were higher in absolute terms in the meshless numerical method when compared with the FEM mesh method, and the NNRPIM presented on average values higher than 6.5%. From these data, we can infer that the used meshless methods are more conservative than the conventional FEM. In addition, through the qualitative evaluation of the stress maps, it is observed that the meshless method presents smoother results, as described in the literature, approaching more in vivo observations, justifying the enthusiasm of the researchers with the use of this methodology.

## Conclusions

Through this study, we can corroborate that the optimal postoperative alignment is within the values that are currently considered of 0 ± 3° varus. It was also observed that the meshless methods obtain smoother and more conservative results, which may make them safer when transposed to the clinical practice. In the future, more studies should be developed using meshless methods, with improvement of the limitations of this study, as well as obtaining results using the 3D model, which will allow to simulate more accurately what is observed in the clinical practice.

## Funding

This research did not receive any specific grant from funding agencies in the public, commercial, or not-for-profit sectors.

## Conflict of interest

The authors declare no conflicts of interest.

## References

[1] OECD. Health at a Glance 2015: OECD Publishing. 2015.

[2] Kurtz S Ong K Lau E Mowat F Halpern M. Projections of primary and revision hip and knee arthroplasty in the United States from 2005 to 2030. J Bone Joint Surg Am. 2007;89:780-785. 10.2106/JBJS.F.00222. 17403800

[3] OECD. Health at a Glance 2011: OECD Publishing. 2011.

[4] Gallo J Goodman SB Konttinen YT Wimmer MA Holinka M. Osteolysis around total knee arthroplasty: a review of pathogenetic mechanisms. Acta Biomater. 2013;9:8046–8058. 10.1016/j.actbio.2013.05.005. 23669623 PMC4003873

[5] Parchi PD Cervi V Piolanti N Ciapin G Andreani L. Densitometric evaluation of periprosthetic bone remodeling. Clin Cases Miner Bone Metab. 2014;11:226–231. 25568658 PMC4269148

[6] Rand JA Trousdale RT Ilstrup DM Harmsen WS. Factors affecting the durability of primary total knee prostheses. J Bone Joint Surg Am. 2003;85-A:259–265. 10.2106/00004623-200302000-00012 12571303

[7] Sikorski JM. Alignment in total knee replacement. J Bone Joint Surg Br. 2008;90-B:1121–1127. 10.1302/0301-620X.90B9.20793. 18757949

[8] Schroer WC Berend KR Lombardi A V. Why are total knees failing today? etiology of total knee revision in 2010 and 2011. J Arthroplasty. 2013;28:116–119. 10.1016/j.arth.2013.04.056. 23954423

[9] Dalury DF Pomeroy DL Gorab RS Adams MJ. Why are total knee arthroplasties being revised ? J Arthroplasty. 2013;28:120–121. 10.1016/j.arth.2013.04.051. 23886410

[10] Kim Y-H Park J-W Kim J-S Park S-D. The relationship between the survival of total knee arthroplasty and postoperative coronal, sagittal and rotational alignment of knee prosthesis. Int Orthop. 2014;38:379–385. 10.1007/s00264-013-2097-9. 24173677 PMC3923934

[11] Fang DM Ritter MA Davis KE. Coronal alignment in total knee arthroplasty. J Arthroplasty. 2009;24:39–43. 10.1016/j.arth.2009.04.034. 19553073

[12] Abdel MP Oussedik S Parratte S Lustig S Haddad FS. Coronal alignment in total knee replacement: historical review, contemporary analysis, and direction. Bone Joint J. 2014;96:857–862. 10.1302/0301-620X.96B7.33946. 24986936

[13] Jeffery RS Morris RW Denham R. Coronal alignment after total knee replacement. J Bone Joint Surg Br. 1991;73:709–714. 1894655 10.1302/0301-620X.73B5.1894655

[14] Lewallen DG Bryan RS Peterson LF. Polycentric total knee arthroplasty. A ten-year follow-up study. J Bone Joint Surg Am. 1984;66:1211–1218. 6490696

[15] Tew M Waugh W. Tibiofemoral alignment and the results of knee replacement. J Bone Joint Surg Br. 1985;67:551–556. https://doi.org/4030849. 4030849 10.1302/0301-620X.67B4.4030849

[16] Parratte S Pagnano MW Trousdale RT Berry DJ. Effect of postoperative mechanical axis alignment on the fifteen-year survival of modern, cemented total knee replacements. J Bone Joint Surg. 92:2010:2143-2149, 10.2106/JBJS.I.01398. 20844155

[17] Morgan SS Bonshahi A Pradhan N. The influence of postoperative coronal alignment on revision surgery in total knee arthroplasty. Int Orthop. 2008;32:639–642. 10.1007/s00264-007-0391-0. 17611758 PMC2551715

[18] Bonner TJ Eardley WGP Patterson P Gregg PJ. The effect of post-operative mechanical axis alignment on the survival of primary total knee replacements after a follow-up of 15 years. Bone Joint J. 2011;93-B:1217–1222. 10.1302/0301-620X.93B9.26573. 21911533

[19] Victor JMK Bassens D Bellemans J Constitutional varus does not affect joint line orientation in the coronal plane. Clin Orthop Relat Res. 2014;472:98–104. 10.1007/s11999-013-2898-6. 23733590 PMC3889437

[20] Daxini SD Prajapati JM. A review on recent contribution of meshfree methods to structure and fracture mechanics applications. Scientific World J. 2014;2014:247172. 10.1155/2014/247172. PMC3910281 24516359

[21] Belinha J Dinis LMJS Jorge RMN. The meshless methods in the bone tissue remodelling analysis. Proced Eng 2015;110:51–58. 10.1016/j.proeng.2015.07.009.

[22] Vanlommel J Luyckx JP Labey L Cementing the tibial component in total knee arthroplasty: which technique is the best?. J Arthoplasty. 2019;26:492–496. 10.1016/j.arth.2010.01.107 20381290

[23] Completo A Fonseca F. Fundamentos de Biomecânica Músculo-Esquelética e Ortopédica. Porto, Portugal: Publindustria, 2011.

[24] Beaupré GS Orr TE. An approach for time-dependent bone modeling and remodeling-application: a preliminary remodeling simulation. J Orthop Res. 1990;8:662–670. 2388106 10.1002/jor.1100080507

[25] Beauprc GS Orr TE Carter DR. An approach for time-dependent bone modeling and remodeling-theoretical development. J Orthop Res. 1990;8:651–661. 2388105 10.1002/jor.1100080506

[26] de Velde SKV Bingham JT Hosseini A Increased tibiofemoral cartilage contact deformation in patients with anterior cruciate ligament deficiency. Arthritis Rheum. 2009;60:3693–3702. 10.1002/art.24965. 19950260 PMC2914513

[27] Proff P Römer P. The molecular mechanism behind bone remodelling: a review. Clin Oral Investig. 2009;13:355–362. 10.1007/s00784-009-0268-2. 19319579

[28] Robling AG Castillo AB Turner CH. Biomechanical and molecular regulation of bone remodeling. Annu Rev Biomed Eng. 2006;8:455–498. 10.1146/annurev.bioeng.8.061505.095721. 16834564

[29] Chapleau J Lambert BS Sullivan TC Clyburn TA Incavo SJ. Impact of valgus vs varus mechanical axis correction during primary total knee arthroplasty on postoperative periarticular bone mineral density. J Arthroplasty. 2021;36:1792–1798. 10.1016/j.arth.2020.12.011. 33384195

[30] Sims NA Gooi JH. Bone remodeling: Multiple cellular interactions required for coupling of bone formation and resorption. Seminars in Cell and Developmental Biology. 2008;19:444–451. 10.1016/j.semcdb.2008.07.016. 18718546

[31] Eckstein F Hudelmaier M Cahue S Marshall M Sharma L. Medial-to-lateral ratio of tibiofemoral subchondral bone area is adapted to alignment and mechanical load. Calcified Tissue Int. 2009;84:186–194. 10.1007/s00223-008-9208-4.Medial-to-lateral. PMC2929533 19148562

[32] Jaroma A Soinninvaara T Kröger H. Periprosthetic tibial bone mineral density changes after total knee arthroplasty periprosthetic tibial bone mineral density changes after total knee arthroplasty A 7-year follow-up of 86 patients. Acta Artop. 2016;87:268–273. 10.3109/17453674.2016.1173982. PMC4900090 27120266

[33] Au AG Raso VJ Liggins AB Amirfazli A. Contribution of loading conditions and material properties to stress shielding near the tibial component of total knee replacements. J Biomech. 2007;40:1410–1416. 10.1016/j.jbiomech.2006.05.020. 16846605

[34] Lonner JH Klotz M Levitz C Lotke PA. Changes in bone density after cemented total knee arthroplasty influence of stem design. J Arthroplasty. 2001;16:12–14. 10.1054/arth.2001.16486. 11172279

[35] Fernandes P Rodrigues H Jacobs C. A model of bone adaptation using a global optimisation criterion based on the trajectorial theory of Wolff. Computer Methods Biomech Biomed Eng. 1999;2:125-138. 10.1080/10255849908907982. 11264822

[36] Nyman JS Hazelwood SJ Rodrigo JJ Martin RB Yeh OC. Long stemmed total knee arthroplasty with interlocking screws: a computational bone adaptation study. J Orthop Res. 2004;22:51–57. 14656659 10.1016/S0736-0266(03)00159-1

